# Frankincense in Material Science and Engineering: Functional Applications, Economic Considerations and Sustainability Perspectives

**DOI:** 10.3390/ma19163402

**Published:** 2026-08-11

**Authors:** Abdullah Al Mashani, Atiya Fatima, Luay Rashan, Alessio Peluso

**Affiliations:** 1 Mechanical & Mechatronics Engineering Department, College of Engineering, Dhofar University, Salalah 211, Oman; 2 Biodiversity Unit, Research Center, Dhofar University, Salalah 211, Oman; afatima@du.edu.om; 3 School of Engineering, Computing and Mathematics, Faculty of Technology Design and Environment, Oxford Brookes University, Headington Campus, Oxford OX3 0BP, UK; apeluso@brookes.ac.uk

**Keywords:** frankincense, *Boswellia*, engineering materials, corrosion inhibition, biomaterials, composite materials, sustainable materials, environmental engineering

## Abstract

Frankincense, a natural resin obtained from *Boswellia* species, has been extensively utilized in traditional medicine and has attracted significant attention in biomedical applications owing to its vast therapeutic effects. While many studies and reviews have reported its pharmacological and biomedical applications, its potential in broader engineering domains and material science remains less explored. In biomedical engineering, frankincense-based compounds have been successfully incorporated into several matrices including hydrogels, nanomaterials, scaffolds, coatings for tissue engineering, food preservation and functional textiles. Frankincense has also exhibited significant potential as a functional component in engineering materials and environmental applications including its significant effects as a green corrosion inhibitor, drilling-fluid modifier and adsorbent materials. This review also highlights the economic aspects of frankincense utilization by evaluating and comparing its production, market value and scalability with other natural resin and gums. Additionally, environmental and sustainability factors associated with frankincense production, harvesting practices and resource availability have also been discussed. Overall, this review provides an overview of frankincense’s potential in diverse engineering applications and highlights the transition of frankincense from a traditional medicinal resin to a promising bio-based material for biomedical, material and environmental engineering applications.

## 1. Introduction

**Figure 1:**
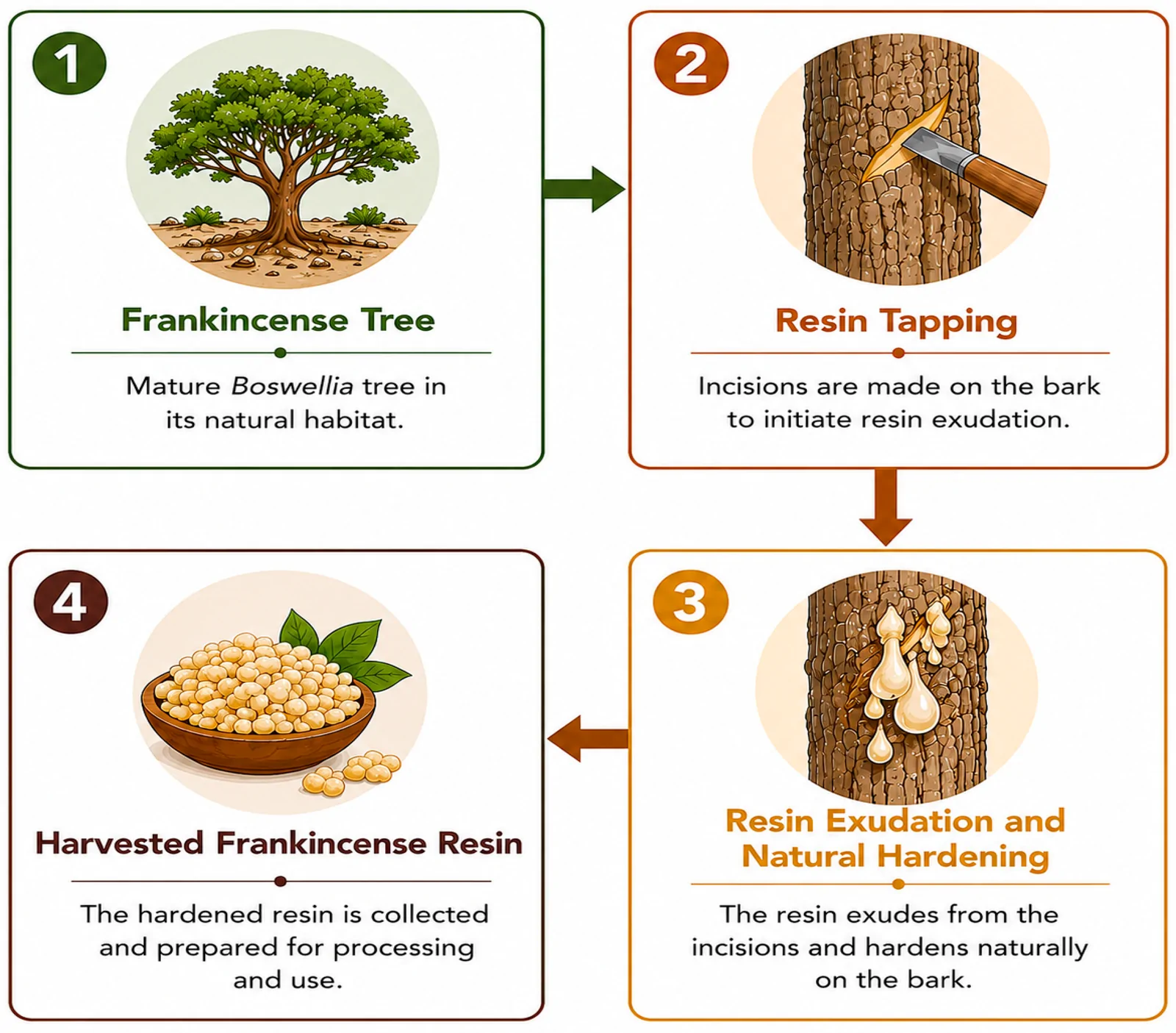
Schematic illustration of the frankincense harvesting process. The illustrations were generated using OpenAI ChatGPT (GPT-5.5, OpenAI) based on an author-developed concept.

Frankincense is a distinctive aromatic gum resin harvested from *Boswellia* trees and has been used for centuries in ancient civilisations for trade, commerce, and religious rituals, making it a captivating natural product [1]. Several *Boswellia* species including *Boswellia sacra* (also called *B. bhaw-dajiana*, *B. carteri*), *B. frereana*, *B. serrata* (*B. thurifera*), and *B. papyrifera* produce frankincense resins. The resin from each species is graded differently depending on harvest timing and is hand-sorted to maintain quality. It is found in the dry mountainous regions of countries such as Yemen, India, China, Somalia, and Oman [2,3,4]. Figure 1 illustrates the stages involved in harvesting frankincense resins from the *Boswellia* tree.

**Figure 2:**
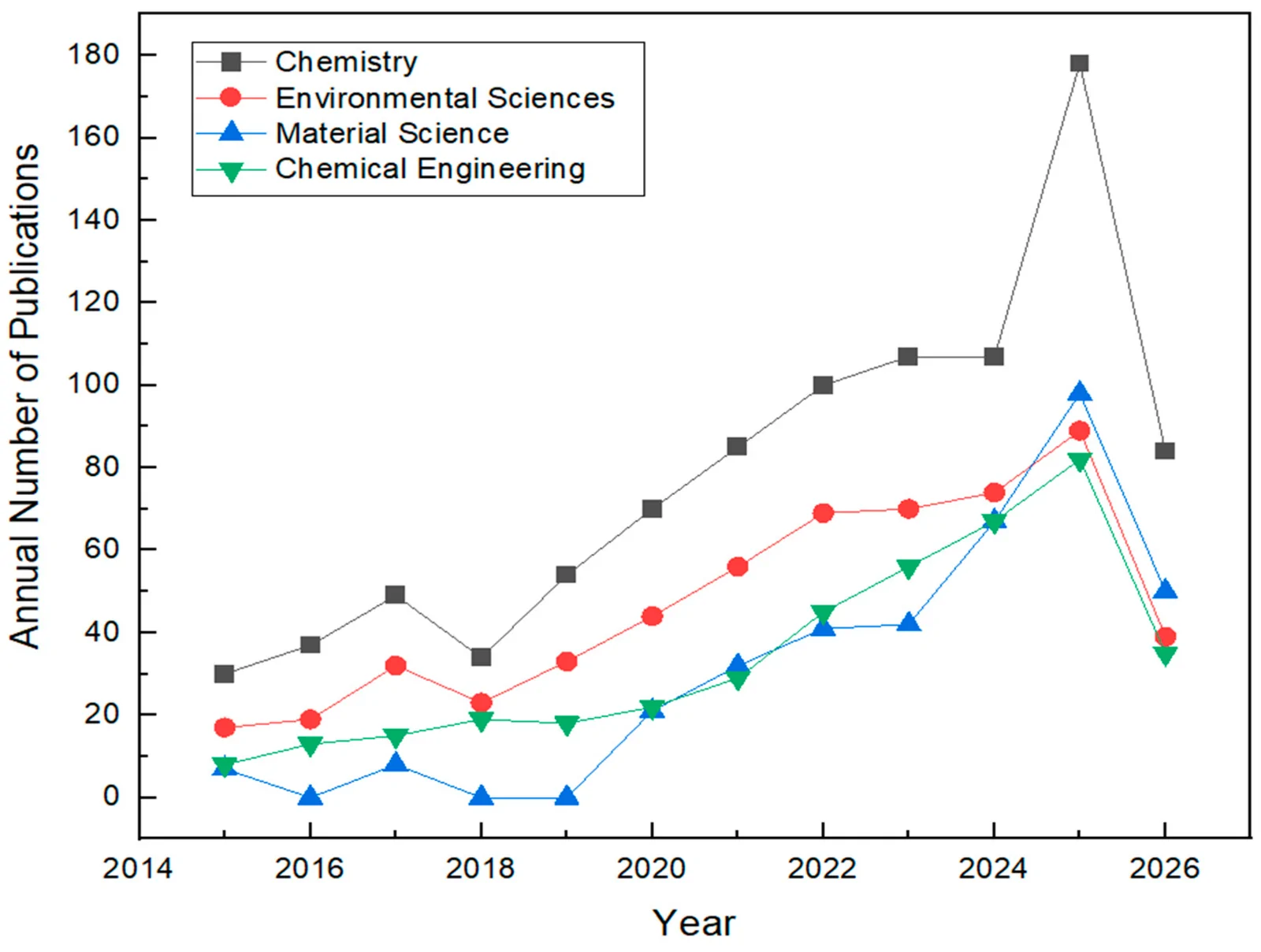
Annual number of publications related to frankincense research in Chemistry, Environmental Sciences, Materials Science, and Chemical Engineering from 2015 to 2026. Data were obtained from the Scopus database.

Frankincense has long been recognized for its therapeutic uses, particularly in medicine, where it has been employed as a remedy for various diseases [5]. It has been used as a treatment for various conditions, including but not limited to cancer, arthritis, asthma, and other inflammatory disorders [6,7]. Recent scientific studies have highlighted the potential of frankincense due to its strong antioxidant and antimicrobial properties [8]. Frankincense has been explored as a resourceful component in developing biomaterials for various bioengineering applications, especially in tissue engineering where it is utilized for creating specialized scaffolds which can be used in developing durable, load-bearing implants such as artificial joints and dental implants [9,10]. Frankincense, as a biomedical material, could help achieve even sustainability targets by minimizing its environmental impact compared to other materials manufactured and processed chemically in factories [11]. As a result, incorporating frankincense into engineering applications, such as biomaterials, has become an important focus, especially within scientific disciplines, due to its natural properties that can be utilized across various engineering fields. Although frankincense has attracted significant attention in the field of biomedical application, its application in other engineering domains remains scarce. Figure 2 demonstrates the use of frankincense over the past decade across various scientific domains.

Numerous studies have examined the biomedical properties of frankincense, particularly its antimicrobial, anticancer, and tissue-regenerative effects. By contrast, limited research has explored its potential in broader engineering fields, including materials, chemical, and environmental engineering, as well as in corrosion protection and industrial technologies. Unlike previous reviews that emphasize biomedical and pharmaceutical aspects, this review adopts a multidisciplinary engineering perspective and critically evaluates emerging applications of frankincense in corrosion protection, drilling fluid enhancement, environmental remediation, advanced functional materials, and sustainable engineering. The review also assesses the current state of knowledge on these engineering applications and examines their underlying mechanisms. It further considers the economic feasibility and sustainability of frankincense from an engineering standpoint, identifies existing limitations, and outlines opportunities for future research and industrial adoption.

## 2. Chemical Composition of Frankincense

**Figure 3:**
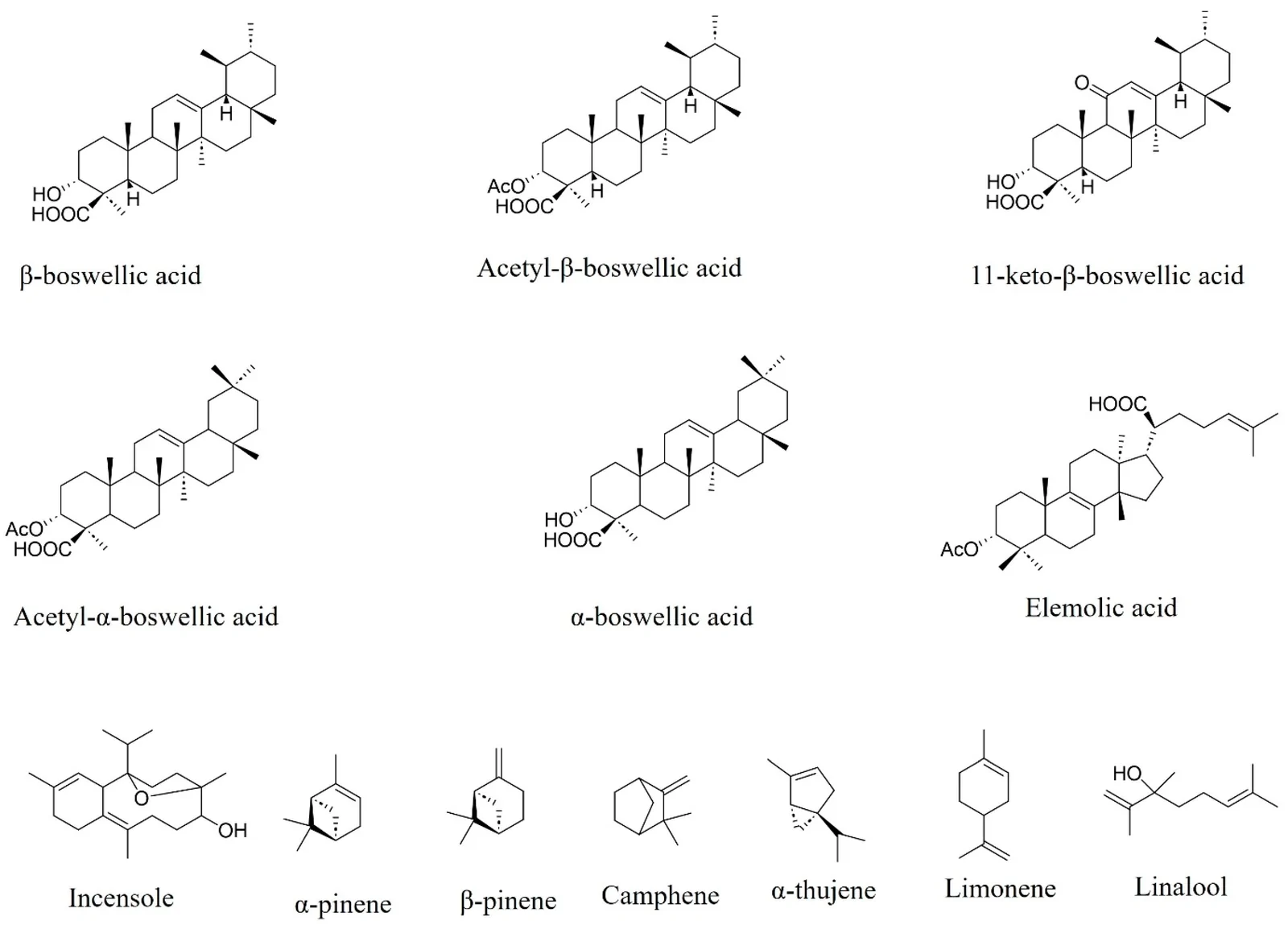
Main chemical constituents of frankincense.

Frankincense resin contains approximately 5–9% essential oil, 65–85% resin, and 21–22% gum. The main chemical constituents isolated from frankincense include pentacyclic triterpenoids, tetracyclic triterpenoids, macrocyclic diterpenoids, and numerous essential oils. Among these, pentacyclic triterpenoids are the most prominent and extensively researched compounds from frankincense. These can be classified as ursolidine, oleanolic, and lupinane, based on their chemical structures. Boswellic acids (BAs), including β-boswellic acid, acetyl-β-boswellic acid, 11-keto-β-boswellic acid (KBA), 3-acetyl-11-keto-β-boswellic acid (AKBA), α-boswellic acid, and acetyl-α-boswellic acid are identified as marker compounds [12]. The chemical structures of the main chemical constituents of frankincense are shown in Figure 3.

The biological properties of frankincense are closely associated with its chemical constituents. BAs are primarily responsible for the anti-inflammatory, anticancer, antimicrobial, antioxidant and hepatoprotective effects of *Boswellia* [13,14]. The multiple hydroxyl and carboxyl functional groups present in its constituents facilitate interactions with proteins, polymers, and metallic surfaces, making them attractive not only for biomedical applications but also for engineering applications. The essential oil fraction, rich in monoterpenes and sesquiterpenes, has been primarily associated with antimicrobial and wound healing properties. Its wound-healing effects are mediated by its anti-inflammatory, antioxidant and anti-apoptotic activities, which facilitate tissue regeneration and wound healing [15].

In engineering applications, boswellic acids and incensole acetate are considered to contribute to corrosion inhibition of stainless steel, although the precise molecular mechanisms remain to be investigated. Frankincense extracts (FE) adsorb onto the metallic surfaces and form a protective layer that inhibits the absorption of chloride ions, thereby improving the corrosion resistance of stainless steel [16]. The constituents of frankincense interact with mineral surfaces and thereby modify the physicochemical properties of clay, which improves the drilling fluid performance [17]. The adsorption performance of frankincense-based materials could be primarily attributed to hydroxyl, carboxyl and other oxygen-containing functional groups, which facilitate hydrogen bonding, electrostatic interactions and surface complexation with pollutants. These interactions play a role in contaminant adsorption and composite stability and support the development of efficient materials for wastewater treatment and other environmental applications.

## 3. Therapeutic Effects of Frankincense

Frankincense has long been recognized as one of the important natural products with potential biomedical applications and has remained an integral part of Chinese, Ayurvedic, African and Middle Eastern medicinal systems. It has been used in traditional medicine in many countries for treating ulcerative colitis, inflammatory diseases and Crohn’s disease [18,19]. Additionally, the essential oil and extracts from frankincense have been utilized in the treatment of asthma, cough and as mouthwash [18]. Currently, extensive studies have been carried out on frankincense to explore its therapeutic efficacy and biomedical applications. The anticancer effects of frankincense are primarily mediated by its essential oil, pentacyclic triterpenes and macrocyclic diterpenes. Among these, BAs (pentacyclic triterpenes) are the most commonly explored for their anticancer activities [13]. Frankincense has shown significant anti-inflammatory effects in numerous studies [14]. The beneficial effects of frankincense in wound healing were investigated through in vivo studies, revealing faster wound contraction, increased epithelialization, and improved collagen synthesis [15].

## 4. Applications

### 4.1. Frankincense in Biomedical Applications

Frankincense has emerged as a promising bioactive component in biomaterials for drug delivery, wound healing, tissue regeneration, dental implants and bone scaffolds owing to its diverse therapeutic properties. The bioactive compounds of frankincense possess antimicrobial, anti-inflammatory, antioxidant properties and aid in tissue regeneration. Consequently, FE, frankincense essential oil (FEO) and bioactive molecules including boswellic acids have been utilized in developing various engineered biomaterial systems such as hydrogels, films, electrospun nanofibers, composite scaffolds and bioactive coatings. These biomaterials exploit the functional characteristics of frankincense and are utilized in healthcare applications and as functional materials.

The use of frankincense in biomaterial engineering has a significant impact, especially when incorporated into glass ionomer cement (GIC), where it improved marginal sealing by reducing the interfacial gap size from 6.88 μm to 5.22 μm without significantly affecting hardness, demonstrating the potential of frankincense as a sustainable bio-based modifier for dental restorative materials [20]. Khadim and Ghalib investigated the effects of adding frankincense as a liquid additive on the thermal conductivity of the GIC, compared with the control material, indicating improved thermal insulation properties. The results suggest that frankincense can serve as a low-cost, environmentally friendly natural additive to modify the thermal performance of dental restorative materials [21].

In tissue engineering, frankincense-based compounds, such as boswellic acids, have been utilized to develop composites (Table 1). Hydrogels based on gellan gum-FE exhibited enhanced chondrogenesis and potential for cartilage healing [22]. Poly ε-caprolactone (PCL)/gelatin scaffolds loaded with boswellic acids displayed potential for the treatment of Parkinson’s disease [23]. Frankincense and graphene oxide along with silicone tube (ST) conduits exhibited significant results in a study conducted to explore their effects in the treatment of nerve injuries [24]. Electrospun nanofibers of carboxymethylated modified frankincense gum (MFG) with Zein-containing peppermint essential oil (PEO) were used to prepare the scaffold Zein/MFG/PEO having antibacterial properties which can be utilized in food product formulation and pharmaceuticals [25]. In another report, frankincense gum was used with tragacanth to develop a multifunctional core–shell nanocomposite (TFH@TA/AuNPs-HDA) with anti-inflammatory and tissue protective properties for their possible use in rheumatoid arthritis (RA) therapy [26]. Mosquito repellent textiles were obtained by Kumar et al. using FEO. Boric acid-chitosan microcapsules were loaded with FEO to develop final fabrics which exhibited excellent mosquito repellency and significant antioxidant and antibacterial activities [27]. Chitosan-FEO based edible coatings protected fresh strawberries from bacterial growth signifying their potential in food preservation [28].

### 4.2. Frankincense in Engineering Materials

#### 4.2.1. Frankincense as a Corrosion Inhibitor

Frankincense is a unique material that can be used in a range of engineering and technical applications. Frankincense acts as an eco-friendly (green) corrosion inhibitor for metals and alloys [29,30]. It has been reported to exhibit strong performance as a corrosion inhibitor on certain steel grades commonly used in industry, demonstrating its effectiveness. Ibrehem et al. tested frankincense as an inhibitor and found it reduced the corrosion rate to below 0.001 under various conditions, including distilled water, salt solutions, and organic solvents [31]. They analyzed carbon steel samples in several solutions: distilled water, salt water with 10 g/L, 20 g/L, and 30 g/L NaCl, and ethanol-water mixtures at different concentrations. Weights were measured over several days to evaluate corrosion, first without coating, then with frankincense coatings, with continued monitoring. The study revealed frankincense to be a highly effective corrosion inhibitor, significantly reducing corrosion rates in multiple environments. These findings suggest strong corrosion resistance, likely due to its chemical composition and inhibitory properties.

Masadeh et al. [16] conducted a study on the effectiveness of frankincense as an environmentally friendly inhibitor of corrosion to prevent pitting corrosion of AISI 304 stainless steel in a 0.5 M ferric chloride (FeCl_3_) solution, using electrochemical noise analysis and potentiodynamic polarization methods. In this study, the following amounts of frankincense were used: 2.5, 5, 7.5 and 10 wt%. It was shown that the greater the inhibitor concentration, the greater the corrosion resistance of the stainless steel. More specifically, the value of corrosion current density changed from 7.17 × 10^−4^ to 4.30 × 10^−4^ A cm^−2^, the highest inhibition efficiency was reached for 7.5 wt% of frankincense (17.31%). The results obtained with the optical microscope confirmed a decrease in the number and size of the corrosion pits. The suggested mechanism of action involves adsorption of frankincense components on the stainless steel surface to form a protective film that reduces the influence of chloride ions and prevents pit formation.

However, the investigation was limited to laboratory exposure in FeCl_3_ solution, and further studies under industrial service conditions and long-term exposure are required before large-scale engineering implementation can be recommended [32].

Table 2 lists the studies that discuss the use of frankincense as an inhibitor in engineering materials.

The findings in Table 2 indicate that frankincense is a promising green corrosion inhibitor for steel and stainless steel across a wide range of corrosive conditions. Most of the corrosion protection arises from the adsorption of bioactive materials (e.g., BAs, terpenoids, and resinous compounds) on the metal surface, which can prevent the penetration of harmful ions (e.g., chloride ions). Compared to standard inorganic corrosion inhibitors, frankincense has the advantages of being biodegradable, low-toxicity, renewable, and having less environmental impact. Yet, in most laboratory-scale experiments, the results have been controlled, and further study is needed to compare the long-term stability, durability, and effectiveness of frankincense in industrial service settings.

#### 4.2.2. Frankincense in Bio-Based Composite Materials

Developments in environmental concerns and regulations have pushed most of the composite industries to revisit natural resources, with a focus on biobased natural fibres to reinforce materials, aiming for low cost, low density, and good mechanical performance [34,35,36].

Temesgen et al. developed a fully biodegradable composite using enset-woven fabric and a bioresin made from equal parts acacia gum and frankincense gum. This bioresin, formulated without synthetic additives, served as the matrix [37]. Optimal mechanical performance was achieved with 30% fabric and 70% bioresin, yielding tensile strength more than four times that of untreated enset fabric, whereas double-layer composites demonstrated approximately twice the strength of single-layer counterparts. The materials also exhibited favourable flexural strength, impact resistance, and thermal stability, indicating their potential for lightweight engineering applications.

These findings demonstrate the potential of frankincense as a renewable bioresin for natural-fibre composites. The good mechanical performance was attributed to effective fibre–matrix bonding, whereas excessive fibre loading promoted void formation and reduced interfacial adhesion, resulting in lower tensile and impact performance. Although the developed composite exhibited promising tensile, flexural and thermal properties for lightweight engineering applications, further research is required to improve moisture resistance, long-term durability, large-scale manufacturing and comparison with conventional epoxy-based systems.

#### 4.2.3. Frankincense in Industrial and Environmental Applications

Frankincense was recently studied as a potential sustainable additive for water-based drilling fluids to improve wellbore stability by inhibiting clay swelling. According to Hosseinzade et al. the performance of FE as a clay-swelling inhibitor was evaluated using sedimentation, rheological, immersion, XRD, FTIR, SEM, particle-size, and zeta-potential studies [17]. The authors found that 5 wt.% FE is effective at inhibiting clay swelling and performs similarly to 3 wt.% potassium chloride (KCl), a commercially available shale inhibitor. Moreover, FE increased the sodium bentonite carrying capacity from 7.5 to 17.5 wt.%, lowered the zeta potential from −24.3 to −5.21 mV, and increased the mean clay particle size from 1034 to 4294 nm, which together suggest efficient inhibition of clay hydration and swelling. XRD and FTIR studies showed that frankincense molecules were incorporated into the interlayer spaces of the clay. The study on drilling fluid compatibility has shown that FE can reduce filtration by acting as a fluid-loss agent. It can be assumed that frankincense is a promising additive for sustainable drilling fluids, given its ability to reduce reliance on conventional inorganic additives in oil and gas drilling. This transformation in the oil and gas sector can help optimize drilling costs during operations.

Furthermore, the use of frankincense has also been reported in water engineering technology. Hussain et al. (2026) developed a frankincense-modified multiwalled carbon nanotube/titanium dioxide (Fr-MWCNTs/TiO_2_) nanocomposite for wastewater treatment applications [38]. The incorporation of frankincense improved the material’s adsorption performance, achieving a methyl orange dye removal efficiency of 96.68% and a maximum adsorption capacity of 709.2 mg/g. The material also exhibited good reusability and stability over multiple adsorption cycles. In a similar study, researchers developed a magnetic nanocomposite composed of frankincense resin, multi-walled carbon nanotubes, and iron oxide nanoparticles that exhibited a high adsorption capacity (322.6 mg/g) for removing amoxicillin from water, demonstrating the potential of frankincense-based materials for wastewater treatment applications [39,40]. Similarly, frankincense gum was utilized to develop a magnetic nanocomposite adsorbent (frankincense-coated carbon nanotubes with iron oxide nanoparticles) capable of efficiently removing toxic Crystal Violet dye from wastewater [41]. Moreover, researchers have successfully converted frankincense soot into fluorescent carbon dots that exhibit high sensitivity toward Pb^2+^ ions, demonstrating their potential as low-cost sensors for water quality monitoring and environmental applications [42]. The study demonstrated that frankincense can serve as a sustainable, bio-based modifier for advanced adsorbents used in environmental engineering and water purification applications.

Collectively, these studies highlight that frankincense can be utilized as a multifunctional bio-based material for industrial, energy, environmental, and advanced engineering applications, as well as traditional biomedical applications. To facilitate comparison among the diverse engineering applications of frankincense, Table 3 outlines the primary application areas, including the materials used, their operating mechanisms, engineering performance, and research challenges. The comparison shows that although frankincense has promising multifunctional capabilities across various engineering fields, most studies remain confined to laboratory settings.

The available research suggests that frankincense has significant potential as a sustainable biomaterial across various engineering fields, including corrosion protection, drilling fluids, wastewater treatment, environmental sensors, and advanced functional materials. However, some critical gaps need attention. Most experiments were conducted under laboratory conditions, with few studies testing the durability and practical application of frankincense-based materials in real industrial settings. Additionally, the chemical composition of frankincense varies depending on *Boswellia* species, geographic origin, harvesting time, and extraction methods. Despite the absence of conflicting findings, differences in methodologies and performance criteria preclude direct quantitative comparisons. Moreover, information on industrial-scale production, economic sustainability, material standardization, life cycle analysis, and regulatory considerations remains lacking. Addressing these issues should be a focus of future research.

## 5. Economic Aspects of Frankincense Usage in Engineering Applications

To assess the feasibility of frankincense as a feedstock for novel scientific and engineering applications, its production and trade should be compared with other natural materials such as myrrh, gum Arabic and copal. With a significant share consumed across many industries worldwide, global trade in frankincense has increased dramatically. Major exporters sell raw resin, essential oils, and processed products to markets around the world, where frankincense is used in perfumes, cosmetics, medicine, and folk medicine. Prices vary by quality, origin, and market demand [4]. High-quality frankincense, particularly from Oman derived from *Boswellia sacra*, commands a higher market value due to its distinct scent and medicinal properties [7,43].

**Figure 4:**
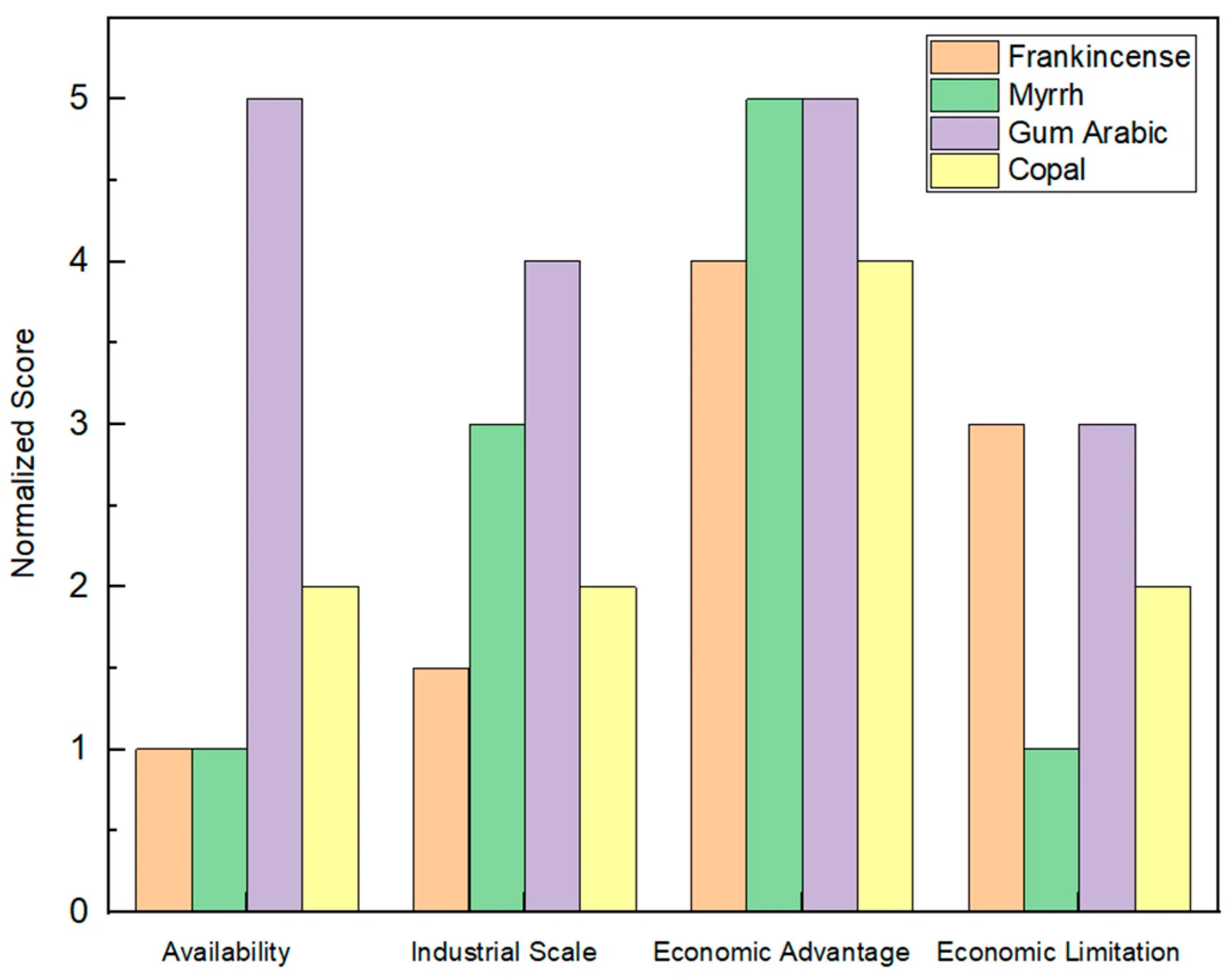
Comparative economic and industrial assessment of frankincense, myrrh, gum Arabic, and copal based on availability, industrial-scale production, economic advantage, and economic limitation (scores assigned from 1 = lowest to 5 = highest).

The cost and availability of frankincense are essential from an engineering viewpoint if it is used as a raw material for new material and bio-based product development. Frankincense can be purchased as crude resin, essential oil, or purified powder, providing several options for different applications [4,42]. Generally, the prices of essential oil and purified extract are higher due to the additional step in purification and processing. Recent data show that the world produces approximately 2000 to 4000 tonnes of frankincense annually, with Somalia accounting for 60 to 70% of this supply. A *Boswellia* tree can yield 1 to 3 kg of resin per season, depending on its health and environmental conditions. The global frankincense market is valued at 10–15 million US dollars per year, and demand continues to increase in the pharmaceutical, cosmetic, and specialized chemical industries [12,44,45,46]. These figures suggest that frankincense could be a valuable natural resource in sustainable engineering practices. However, to ensure its long-term use, it is essential to harvest it responsibly and protect *Boswellia* trees to prevent a decline in supply [45]. Table 4 presents a comparative analysis of the economic and production characteristics of natural resins and gums. Figure 4 depicts a bar chart showing natural resins and gums classified by availability, market size, value per unit mass, scalability, and cost efficiency, derived from Table 4. It describes a comparison between frankincense, myrrh, gum Arabic, and copal across availability, industrial scalability, economic advantages, and economic constraints. Gum Arabic recorded the highest values for availability, industrial scalability, and economic advantages, confirming its strong position in commerce and industry. Myrrh showed moderate values for industrial scalability and economic benefits; however, its geographical availability is a limiting factor. Copal recorded moderate values, indicating that industrial use is quite limited. Frankincense has unique characteristics, including limited availability and limited industrial scalability, but offers significant economic advantage due to its premium market value and specialized applications.

## 6. Environmental Perspectives of Frankincense Usage in Engineering Applications

**Figure 5:**
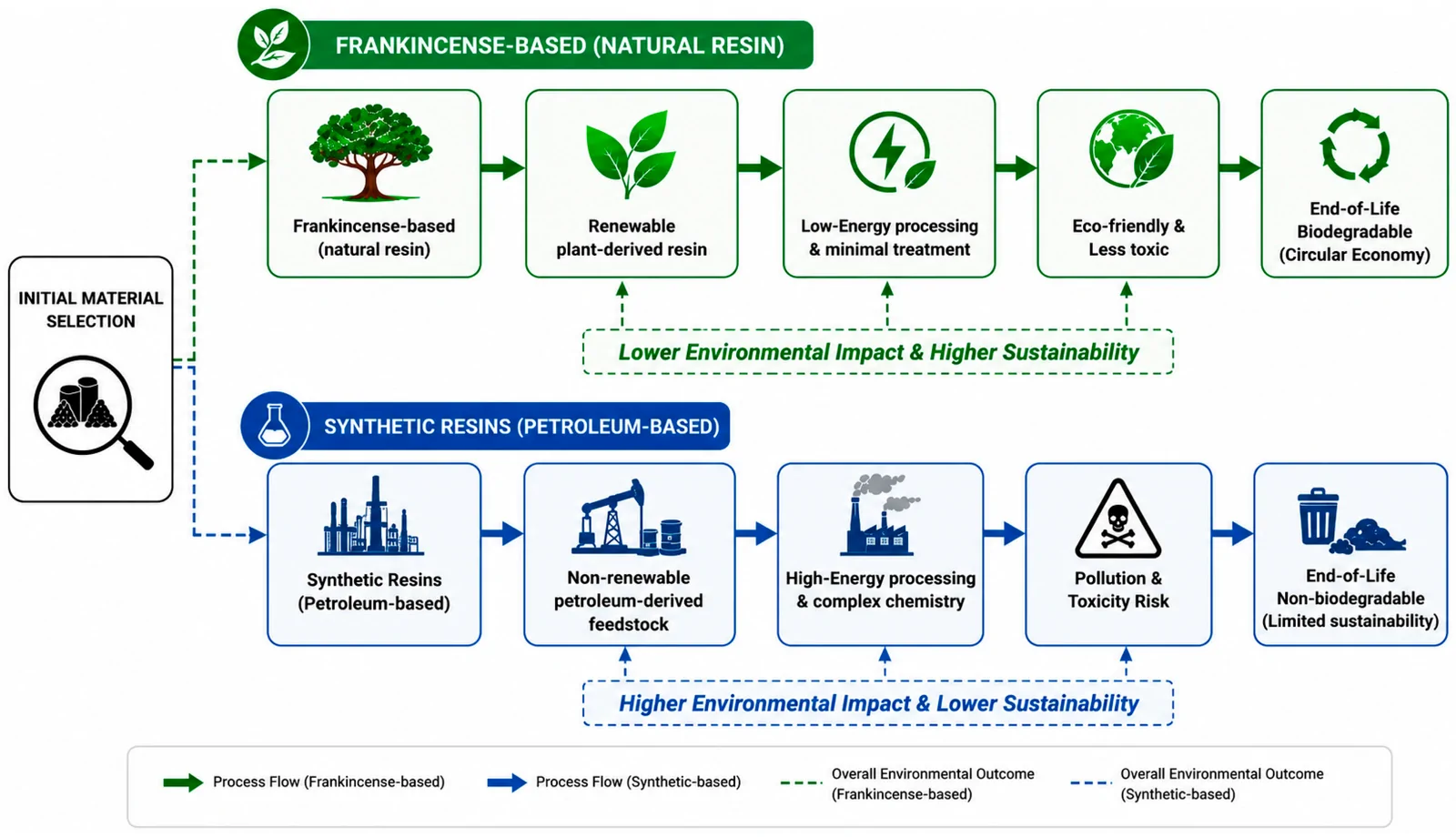
Comparative environmental pathways of frankincense-based and synthetic resin materials. The schematic illustrations were generated using OpenAI ChatGPT (GPT-5.5, OpenAI) based on an author-developed concept.

Increasing interest is being shown in bio-based materials as sustainable alternatives to petroleum-based polymers as they are associated with problems, such as a higher carbon footprint and poor biodegradability [50,51]. In this context, natural resins, such as frankincense, have garnered significant attention as they are directly extracted from plants, do not require excessive treatment, and are fully biodegradable. Although frankincense-based materials are biodegradable and derived from natural resources, their overall environmental sustainability depends on responsible harvesting, traceable supply chains, and effective conservation of *Boswellia* species. Figure 5 compares life-cycle sustainability between frankincense-based natural resins and conventional petroleum-based synthetic resins.

In addition, the actual tapping practices of frankincense resins may compromise the reproductive capacity of *Boswellia* trees and put them at risk of extinction, if sustainable management is not implemented to protect biological resources [52]. According to analysis by Mekoya and Adgo, excessive harvesting of the resins impedes natural regeneration, resulting in a *Boswellia* tree population dominated by mature plants [53]. Fadul et al. identified other causes, such as the expansion of other cultivations, illicit cutting, and animal grazing, as the main production constraints [54]. Birhan et al. documented a decline in production and in export volumes in Ethiopia [55], while DeCarlo et al. reported a similar situation in Somaliland [56]. Furthermore, Mekoya and Adgo highlighted the fact that the ecological role of *Boswellia papyrifera* in combating desertification is also threatened by the ongoing decline of its natural population [53].

The relationship between trade and conservation is complex, and they often play antagonistic roles. Johnson and Ablard noted that the economic value of trees can protect land from conversion, while high demand drives overharvesting of the same trees [57]. Johnson et al. argued that the international Plants Committee of the Convention on International Trade in Endangered Species of Wild Fauna and Flora (CITES) listing is ill-suited to conserving *Boswellia* species due to limited infrastructure and corruption in exporting states, suggesting that regulations in importing countries may be more effective at driving private supply chain improvements [58]. Tesfay et al. concluded that the current production and export system is unsustainable, a finding also reported by DeCarlo et al. who highlighted that rising demand has led to unsustainable harvesting without improving community livelihoods [56,59].

DeCarlo et al. [56] attributed unsustainability to short-term incentives and the poverty trap, documenting that there was a substantial increase in demand and resin prices between 2010 and 2017, which thereby pushed harvesters towards unsustainable practices to take advantage of the rising prices. Fadul et al. noted that resin quality is compromised because gum processing and storage are carried out by unskilled personnel [54]. Several authors have proposed various strategies to improve sustainability, such as regulated tapping, controlled grazing, ex situ conservation [53], training and capacity building for local communities [54,60], and finally the introduction of new technologies for the harvest [56].

According to several reports, several factors need to be considered when utilizing frankincense in engineering applications. Additional demands could intensify the documented overharvesting and population decline of *Boswellia* trees. Moreover, the lack of traceability prevents verification of the frankincense’s sustainable sourcing [56,57].

Additional demand from such applications could exacerbate the documented overharvesting and decline of *Boswellia* populations. The profitability of the supply chain for all actors, documented by Fadul et al. suggests that increased demand could intensify existing pressures. The lack of traceability, highlighted by Johnson and Ablard and DeCarlo et al., currently prevents verification of whether frankincense used in engineering applications comes from sustainable sources [54,56,57]. The unsustainability of the current system, as concluded by Tesfay et al., implied that labelling frankincense as an “ecological” material for engineering uses would require independent verification of supply chain practices [59].

Use-phase and end-of-life factors also matter for each engineering application, beyond supply-side considerations. The literature on the subject is scarce and mainly focused on incense combustion, documenting emissions of volatile organic compounds and particulate matter [61,62,63,64]. Toxicological studies indicate low acute toxicity of boswellic acids [65], although very high doses have been associated with hepatotoxicity [66,67]. However, the literature on the environmental impacts of incorporating frankincense into engineering materials is lacking, representing a priority area for future research.

## 7. Limitations and Practical Challenges

Despite the increasing interest in frankincense as a natural product for engineering applications, various limitations restrict its widespread applications. One of the major challenges associated with frankincense resin is variation in its chemical composition due to differences in origin across *Boswellia* species, geographical location, tree age, harvesting period, and extraction techniques. Since the concentrations of key constituents such as terpenes and BAs may vary considerably, achieving stable material performance becomes difficult. Additionally, these factors also pose a challenge in standardization, quality control and reproducibility of frankincense-based engineering materials.

Another important factor is the limited understanding of the structure-property relationship between frankincense-based materials. While many studies have reported enhancements in corrosion resistance, antimicrobial properties, and adsorption capacities, the mechanisms by which these materials interact with polymeric substances, metallic substrates, and nanocomposites remain poorly explored. Moreover, studies regarding the stability and durability of frankincense-based biomaterials are also inadequate. Most of the research has focused on short-term laboratory-scale evaluations, whereas engineering applications require materials that can withstand prolonged exposure to various environmental conditions, including thermal, chemical, and mechanical stresses.

Furthermore, biomedical and healthcare materials largely depend on the controlled release kinetics of active molecules and the mechanical integrity of the developed composites. Additionally, the use of FEO poses another hindrance due to its volatility. Cytotoxicity, biocompatibility and regulatory evaluations are also required to achieve large-scale application of these composites in the healthcare industry.

In engineering applications, studies have been conducted on a small scale, with limited reports on industrial environments. For instance, although certain studies have reported high inhibition efficiencies in corrosion inhibition of frankincense, their performance under complex industrial conditions such as fluctuating temperature, pressure and flow conditions has not been fully investigated. Similarly, in heavy metal adsorption, the adsorption films studied may exhibit reduced stability in harsh, large-scale operational plants. Similarly, although frankincense has shown promise as a renewable bioresin, its application in bio-based composite materials remains in its early stages of development. Further research is required to address challenges associated with fibre–matrix compatibility, moisture resistance, long-term durability, scalable manufacturing, and commercial implementation. Future studies should also evaluate the performance of frankincense-based composites under different environmental and mechanical loading conditions and benchmark them against conventional epoxy-based composite systems to determine their suitability for broader engineering applications. Also, information on the economic aspects, large-scale manufacturing, and industrial feasibility of frankincense-based materials remains scarce. Finally, the availability of frankincense resin and its sustainable exploitation for large-scale applications pose significant barriers. Widespread engineering applications could require resin beyond traditional markets, increasing the need for improved, sustainable harvesting practices.

## 8. Conclusions and Future Aspects

Frankincense has been used in traditional medicine and has gained significant attention for its therapeutic efficacy. It has also emerged as a promising bio-based material in engineering applications, although the level of experimental validation varies across different fields. According to studies, frankincense possesses excellent biological properties, chemical properties and mechanical characteristics, which have enabled its incorporation into a wide range of materials for varied applications. Among these, biomedical applications are supported by substantial experimental evidence, with FE, essential oils, and key molecules such as BAs having been successfully combined with polymers, gums, etc., to develop hydrogels, films, scaffolds, and nanomaterials for tissue engineering and other functional applications. Several *Boswellia* extracts and BA-based formulations have progressed to clinical studies, and several commercial products are already available in the market for therapeutic use [68,69]. However, clinical translation and commercialization of these materials remain limited.

Apart from biomedical applications, frankincense has also been utilized in developing sustainable engineering materials. Experimental studies have demonstrated its application as a green corrosion inhibitor for stainless steel systems. Recent reports have explored its effects on drilling fluid, dental restorative materials, and wastewater treatment. These findings highlight the potential of frankincense as a multi-functional component in bio-based materials. However, applications of frankincense in these domains remain in their early stages of development and require further validation under practical conditions.

Certain factors, such as variation in chemical composition, inadequate studies on structure-property relationships, limited data on long-term durability, and a lack of standardized processing techniques, restrict its broad-scale application. Furthermore, the limited availability of frankincense and concerns regarding overharvesting of frankincense resins demand careful resource management prior to large-scale commercialization.

While several biomedical and corrosion-protection applications are supported by experimental studies, many other engineering applications remain in the laboratory stage and require further validation. Pilot-scale validation under realistic conditions, along with comprehensive long-term durability and toxicity assessments, is required before wide-scale industrial-level implementation can be achieved. Future studies should focus on standardizing frankincense resin composition and establishing standardized protocols for material reproducibility. Specific attention should be given to the development of advanced composites, smart coatings, anti-corrosive systems and hybrid nanomaterials incorporating frankincense-based compounds. In biomedical applications, more studies are required on controlled release of active molecules/drugs, long-term biocompatibility, and clinical translation studies for successful clinical transition of composites. From a sustainability perspective, studies on life-cycle assessment, economic analysis, and supply-chain management should be pursued to ensure the efficient utilization of resources.

## Figures and Tables

**Table 1:** Frankincense-based materials for biomedical and functional applications.

S. No.	Frankincense-Based Compounds	Material System	Functional Effects	Application	Ref.
1	FE	Gellan gum hydrogels	Enhanced chondrogenesis	Cartilage tissue engineering	[22]
2	FE	Silicone tube conduits	Increased axonal regeneration and myelin formation	Nerve tissue engineering	[24]
3	Carboxymethylated modified frankincense gum	Electrospun nanofiberous scaffolds	Antibacterial properties	Food product formulation and pharmaceuticals	[25]
4	FEO	Boric acid-chitosan microcapsules	Mosquito repellency, antioxidant, antibacterial activities	Mosquito repellent textiles	[27]
5	FEO	Chitosan-based edible coatings	Antibacterial effects	Food preservation	[28]

**Table 2:** Summary of previous studies on the use of frankincense as a corrosion inhibitor for engineering materials.

S. No.	Frankincense-Based Compounds	Material System	Functional Effects	Mechanism	Ref.
1	FE	Steel	Corrosion decreased	Surface adsorption	[31]
2	FE	Carbon steel	Improving inhibition performance to 87.2%	Adsorption	[33]
3	Frankincense resin	Low Carbon Steel	Providing corrosion protection efficiencies exceeding 95%	adsorption and protective film formation on the metal surface	[32]
4	Frankincense resin	Stainless steel AISI 304 SS	Pitting corrosion was significantly reduced	Formation of a protective film on the steel surface	[16]

**Table 3:** Summary of industrial and environmental engineering applications of frankincense-based materials.

S. No.	Application	Frankincense-Based Material	Material System	Mechanism	Performance	Current Limitation	Ref.
1	Corrosion inhibition	Frankincense extract/resin	Carbon steel, Low-carbon steel, AISI 304 stainless steel	Adsorption and protective film formation	Enhanced pitting corrosion resistance, achieving a maximum inhibition efficiency of 17.31%.	Mostly laboratory studies	[16]
2	Drilling fluids	Frankincense extract	Sodium bentonite	Clay swelling inhibition	Comparable performance to KCl	Limited field validation	[17]
3	Pharmaceutical wastewater treatment	Magnetic frankincense–MWCNT nanocomposite (Fr@MMWCNTs)	Amoxicillin-contaminated water	Adsorption	Maximum adsorption capacity of 322.6 mg/g; rapid adsorption equilibrium (25 min); Langmuir isotherm and pseudo-second-order kinetics.	Adsorbent performance declined after three reuse cycles; large-scale application and long-term stability require further investigation.	[39]
4	Heavy metal removal	Frankincense-coated MWCNT/Fe_3_O_4_	Cu^2+^-contaminated water	Adsorption	250 mg/g Cu^2+^ adsorption capacity; effective removal at pH 6–8; Langmuir adsorption model.	Laboratory-scale study: regeneration and long-term performance require further investigation.	[40]
5	Dye removal	Frankincense-coated CNT/Fe_3_O_4_	Crystal Violet wastewater	Adsorption	Efficient dye removal	Continuous-operation stability unknown	[41]
6	Environmental sensing	Frankincense-derived carbon dots	Pb^2+^ detection	Fluorescence sensing	High sensing sensitivity	Early-stage development	[42]

**Table 4:** Economic Characteristics of Frankincense Compared with Other Natural Resins.

Material	Availability	Approximate Market Value	Industrial Scale Production	Economic Advantage	Economic Limitation
Frankincense	Limited	Global market 10–15 million USD [7]	Low	High-value natural resin with niche market demand	Limited supply and dependence on tree harvesting
Myrrh	Limited–moderate (same regions as frankincense)	$260 million in 2025 [47]	Medium	Moderate-value resin used in pharmaceuticals, cosmetics, and traditional medicine	Limited geographical availability and less industrial scalability
Gum Arabic	Moderate–high	USD 1.1 billion in 2025 [48]	High	widely used industrial hydrocolloid with high demand in the food, pharmaceutical, and cosmetic industries	Low value addition in producing countries and dependence on raw material export
Copal	Moderate (Latin America, Africa)	~Approximately USD 500 million in 2023 [49]	Low	Low-cost natural resin used in varnishes, coatings, and traditional applications	Limited high-value industrial applications and fragmented market structure

## Data Availability

No new data were created or analyzed in this study. Data sharing is not applicable to this article.
